# Development of an active packaging based on polyethylene containing linalool or thymol for mozzarella cheese

**DOI:** 10.1002/fsn3.2334

**Published:** 2021-05-19

**Authors:** Shadi Chang, Abdorreza Mohammadi Nafchi, Homa Baghaie

**Affiliations:** ^1^ Food Science and Technology Department Food Biopolymer Research Group Damghan Branch Islamic Azad University Damghan Iran; ^2^ Food Technology Division School of Industrial Technology Universiti Sains Malaysia Penang Malaysia

**Keywords:** active packaging, antimicrobial activity, linalool, mold and yeast, thymol

## Abstract

The aim of this research was to evaluate the effect of active polyethylene film (PE) containing linalool and thymol active components on the microbial shelf life of mozzarella cheese. PE films containing different concentrations of linalool or thymol (0%, 1%, 1.5% and 2%) were prepared. The antimicrobial properties of the films were examined, and mozzarella cheese was packed with these active films. The antimicrobial properties of packed samples during 30 days of storage were studied. The obtained results from film tests showed that by increasing the concentration of active agents (linalool and thymol) in PE films, the antimicrobial activities of film samples against *Escherichia coli*, *Staphylococcus aureus*, *Listeria innocua,* and *Saccharomyces cervicea* were increased. The cheese tests result demonstrated that mozzarella cheese packaging with PE films containing different concentrations of linalool and thymol leads to a decreased growth rate of molds and yeasts in cheeses. At the end of the storage period, the lowest number of molds and yeasts was for a sample packed in PE film containing 2% thymol, which increased from 1.00 to 1.21 Log CFU/g during the storage period. From *E. coli* and *S. aureus* contamination, the samples packed in active films were safe until the last day of storage (30th day), while the control sample was unacceptable at 17th day of storage. According to obtained results from this study, it was concluded that the addition of linalool and thymol active components to PE film had a positive effect on the extension of the mozzarella scheese shelf life.

## Graphical abstract

The effect of active polyethylene film (PE) containing linalool and thymol active components on the microbial shelf life of mozzarella cheese was investigated. The antimicrobial activity of film samples against *Escherichia coli*, *Staphylococcus aureus*, *Listeria innocua*, and *Saccharomyces cervicea* was proved. The addition of linalool and thymol active components to PE film had a positive effect on the extension of the mozzarella cheese shelf life.

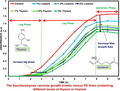

## 1 INTRODUCTION

Mozzarella cheese is one of the most important types of cheese, classifying into two general categories based on its moisture content, including cheese with low moisture content (45%–54%) and cheese with high moisture content (56%–65%). Mozzarella cheese with low moisture content is known as pizza cheese. With a wide variety of microorganisms found in this product, Mozzarella cheese has a relatively short shelf life (Altieri et al., 2005). This type of cheese is classified as perishable food because it can only be refrigerated for about 30 days (Jafarzadeh et al., 2019). In the food industry, packaging systems are used to protect packaged foods during storage and handling (Garavand et al., 2020; Ribeiro‐Santos et al., 2017).

Today, one of the most critical issues in the food industry is maintaining quality, increasing safety, and extending the shelf life of food products (Jahdkaran et al., 2021). Foods can be affected by various chemical and microbial spoilages. These destructive processes can cause the quality of food products to deteriorate and reduce the organoleptic quality and nutritional value of products. In these decaying products, pathogenic microorganisms can also grow (Belasli et al., 2020). In this case, the food products become inedible. Microbial contamination of foodstuffs may occur during various stages such as food production and processing, transportation, or distribution. Since plastics have a low cost, easy processing, and availability, they are most used in the food packaging industry (Agrillo et al., 2019; Mousavian et al., 2021).

One way to control microbial and fungal growth in food products and prevent foodstuff spoilage is to use active antimicrobial packaging (Ekramian et al., 2021; Moslehi et al., 2021; Vilas et al., 2020). Antimicrobial packaging plays a crucial role in developing the safety and food products shelf life. These packages can improve food quality and safety by increasing the lag phase and preventing the growth of microorganism (Motelica et al., 2020). In the most common type of antimicrobial packaging, the active antimicrobial agent is incorporated into the packaging and is not in direct contact with the food (Jafarzadeh et al., 2021; Motelica et al., 2020; Toscano Ávila et al., 2020). In these packaging systems, the release of antimicrobial agents often occurs in a controlled manner, and a suitable concentration of these substances is always available to prevent microbial growth (Galotto et al., 2012; Jafarzadeh & Jafari, 2020; Moshe Dvir et al., 2019).

Essential oils derived from plant sources show significant and effective antimicrobial and antifungal activities and can be used as an alternative to chemical preservatives in antimicrobial food packaging (Suppakul et al., 2011; Xue Mei et al., 2020). Various studies have been performed on the biological activities of essential oils obtained from multiple sources, and these essential oils and their active compounds have been used successfully in antimicrobial packaging systems (Hassan & Cutter, 2020; Li et al., 2020; Moshe Dvir et al., 2019; Tonyali et al., 2020).

Thymol, with the scientific name of 2‐isopropyl‐5‐methylphenol, is an important monoterpenoid compound in some essential oils with a single phenolic ring in its structure and is composed of the connection of two isoprene molecules and three functional groups (Peixoto‐Neves et al., 2010). The presence of functional groups (hydroxyl groups) in the thymol structure plays a significant role in the functional activities of this compound, such as antioxidant and antimicrobial activities. The safety of thymol and its use as a food additive has been approved by the Federal Drug Administration (FDA) (Kachur & Suntres, 2020). Various scientific studies have shown that thymol has significant antimicrobial activity against both gram‐negative and gram‐positive bacteria (Kachur & Suntres, 2020; Khairuddin et al., 2020; Oussalah et al., 2007).

Linalool (3,7‐dimethyl‐octa‐1,6‐dien‐3‐ol) is another important compound found in the essential oils of some plants, which is terpenic alcohol and belongs to the monoterpene family (Aprotosoaie et al., 2014; Dutra et al., 2016). Linalool is an aromatic compound used in the pharmaceutical, perfumery, cosmetics, and food industries (Lapczynski et al., 2008). The US Food and Drug Administration has recommended linalool as a safe food compound (Hsu et al., 2013). The antimicrobial activity of linalool has been confirmed in various studies (Klein et al., 2013; Liu et al., 2020; Prakash et al., 2019).

To the best of our knowledge, active PE films containing natural active compounds from herbal sources have not developed dairy product shelf life. Therefore, in this study, the antimicrobial activity of PE films containing different concentrations of thymol and linalool was investigated. These films were used to extend the shelf life of mozzarella cheese.

## 2 MATERIALS & METHODS

### 2.1 Materials

Polyethylene (PE) film was prepared from Sultan Chap Co., with thicknesses of 0.02 ± 5.7 × 10^–4^ (mm). Thymol and linalool (CAS 64‐17‐5 EMSURE^®^ Reag. Ph Eur, Merck), and Mueller Hinton Broth (MHB), Nutrient Broth, and Baird‐Parker agar were purchased from the Merck Company. *E. coli* O157:H7, *S*. *aureus* ATCC 6538*, Listeria innocua, and Saccharomyces cervicea* were also collected from the central laboratory of the Scientific and Industrial Research Organization (Tehran, Iran). All other chemicals used were of analytical grade.

### 2.2 Preparation of PE films containing thymol and linalool

To prepare film samples, film sheets based on PE resins were crushed by an industrial shredder and turned into powder. It was then combined directly with different concentrations (0%, 1.0%, 1.5% and 2.0%) of antimicrobial agents (thymol or linalool) and mixed well at room temperature to form a uniform form. The desired PE films were made using an intensive molding method with a thickness of 2 mm. After the compression operation, the produced films were immediately placed in an aluminum foil to prevent the loss of antimicrobial agents (Mistry, 2006).

### 2.3 Determination of the kinetic model of microbial growth in dynamic mode

For this purpose, standard microbial strains of *E*.* coli, Staphylococcus aureus*, *Listeria innocua,* and *Saccharomyces cervicea* were prepared as lyophilized from the central laboratory of the Scientific and Industrial Research Organization of Iran. Microbial ampules were carefully broken under sterile conditions under a microbial hood. Then 0.5 ml of sterile Nutrient Broth culture medium was pipetted into the ampule and stirred with a needle. The microbial suspension was then poured into a container containing a sterile culture medium and kept at 37°C for 24 hr. Four regions were cultured linearly in Mueller‐Hinton agar medium and were incubated at 37°C for 24 hr (Akbariazam et al., 2016).

The flask method was used to evaluate the antimicrobial effect of PE films containing thymol and linalool on the studied microorganisms by a dynamic method. To perform the rotating flask method, eight samples of PE films were placed in 100 ml of sterile Nutrient Broth medium for bacteria at a rotational speed of 150 rpm for 23 min. It was then placed in a rotary incubator at 37°C. Since the microbial population is directly related to light density, the adsorption intensity curve based on time was used to show the dynamic microbial growth trend. Microbial growth results were obtained by reading the adsorption changes at 600 nm using a spectrophotometer (Jenway 6305) for 12 hr at regular intervals of 1 hr (Zwietering et al., 1990).

### 2.4 Investigating the effect of PE films containing thymol and linalool on the preservation of mozzarella cheese

#### 2.4.1 Preparation of packaged mozzarella cheeses

Low‐moisture mozzarella cheese was purchased from the factory (Pegah) and divided into different batches of 150 g. Each of these pieces of cheeses was packed with PE films containing different concentrations of thymol and linalool and then stored at refrigerator temperature (4°C) for 30 days. Microbial tests were performed on cheese samples on days 0, 7, 17 and 30 of storage.

#### 2.4.2 Microbial evaluation of cheeses

Mold and yeast colonies were counted with yeast glucose chloramphenicol agar following the Iranian National Standard No. 10154 during the cold storage. Plates were incubated under aerobic conditions at 25 ± 1°C for 5 days; the colonies were counted after incubation.

*Escherichia coli* colonies were counted with lauryl sulfate tryptose broth and EC broth following the Iranian National Standard No. 5234 during the cold storage. Tubes were incubated under aerobic conditions at 44°C for 48 hr; the colonies after the end were counted for incubation.

*Staphylococcus aureus* colonies were counted with Baird‐Parker agar following the Iranian National Standard No. 6806 during the cold storage. Plates were incubated under aerobic conditions at 37 ± 1°C for 48 hr; the colonies were counted after incubation.

#### 2.4.3 Statistical analysis

The statistical analysis of obtained data was carried out using analysis of variance by IBM SPSS Statistics 22.0 (SPSS, Inc.,). Comparisons between samples were analyzed using Duncan multi‐range test. Differences were considered to be significant at *p* < .05.

## 3 RESULTS AND DISCUSSION

### 3.1 Investigation of antimicrobial activity of PE films by dynamic method

The microbial growth curve has four phases based on time. In the first phase (lag phase), the microorganisms adapt to the environment and are ready to grow. In the second stage (log phase), the microbial cell begins to multiply logarithmically, and its population increases. In the third stage (the constant growth phase), the environmental conditions and the amounts of food for the microorganism are limited. The production population is equal to the dead population, in which case the total number of microorganisms remains constant. In the last stage (death phase), after the break in the constant phase, if there is not enough food for the microorganisms, their population will decrease.

**FIGURE 1:**
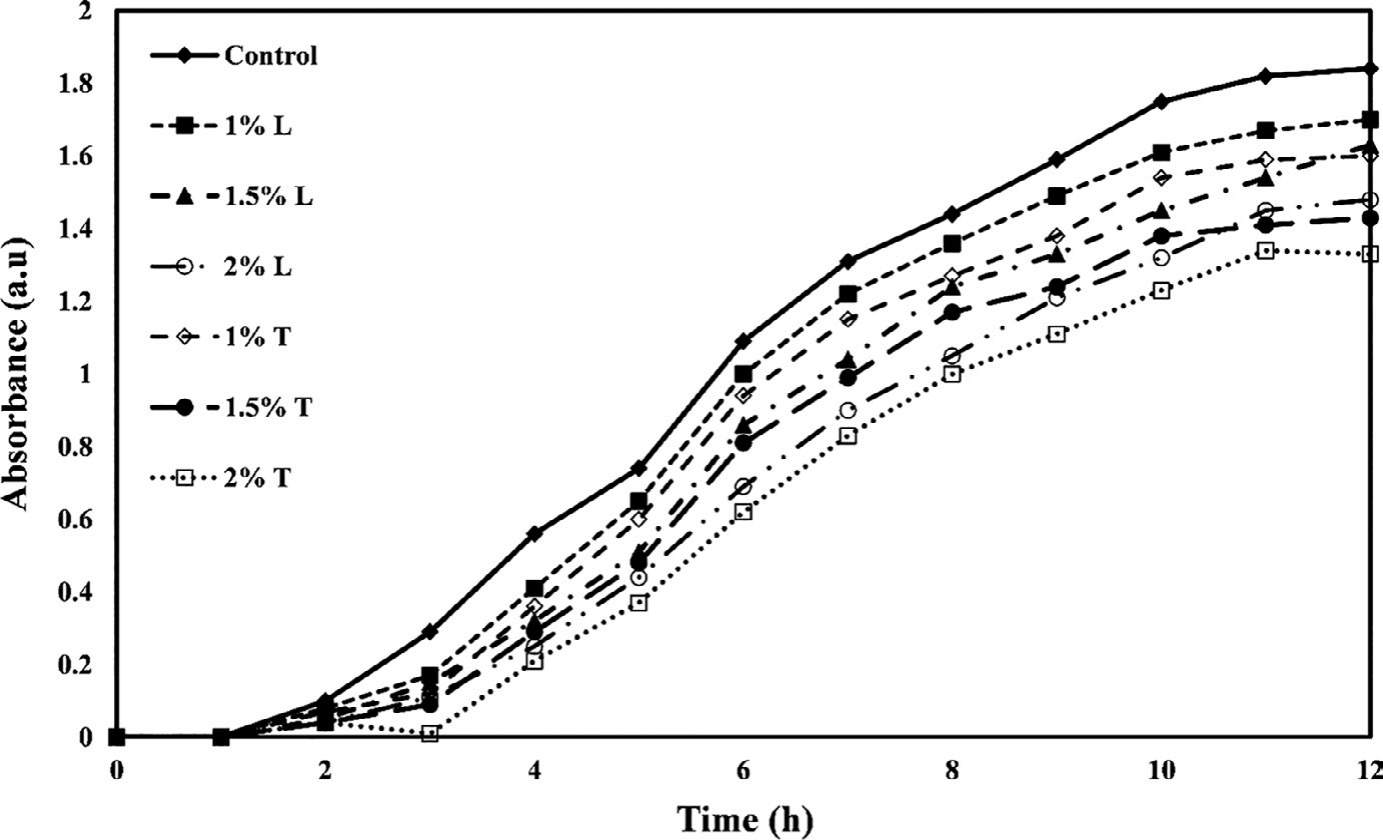
*Escherichia coli* growth kinetic versus PE films containing different levels of thymol (T) or linalool (L)

**FIGURE 2:**
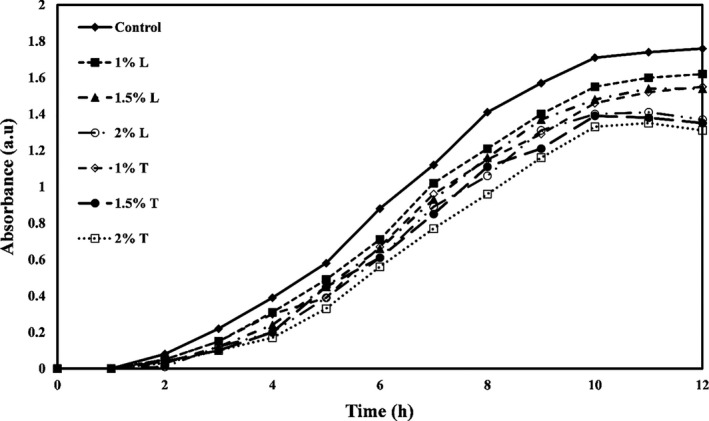
*Staphylococcus aureus* growth kinetic versus PE films containing different levels of thymol (T) or linalool (L)

**FIGURE 3:**
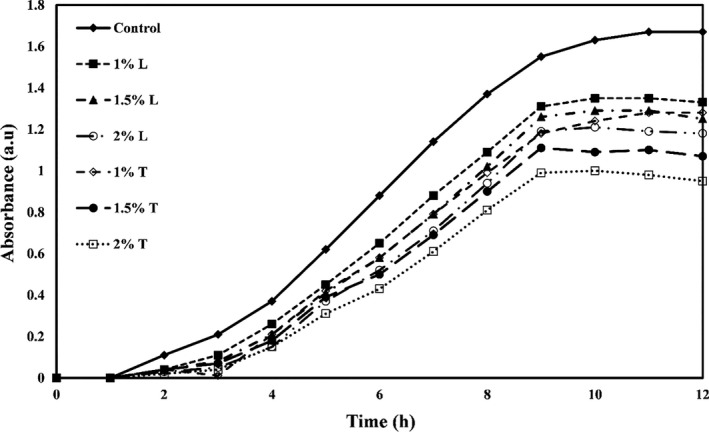
*Listeria innocua* growth kinetic versus PE films containing different levels of thymol (T) or linalool (L)

**FIGURE 4:**
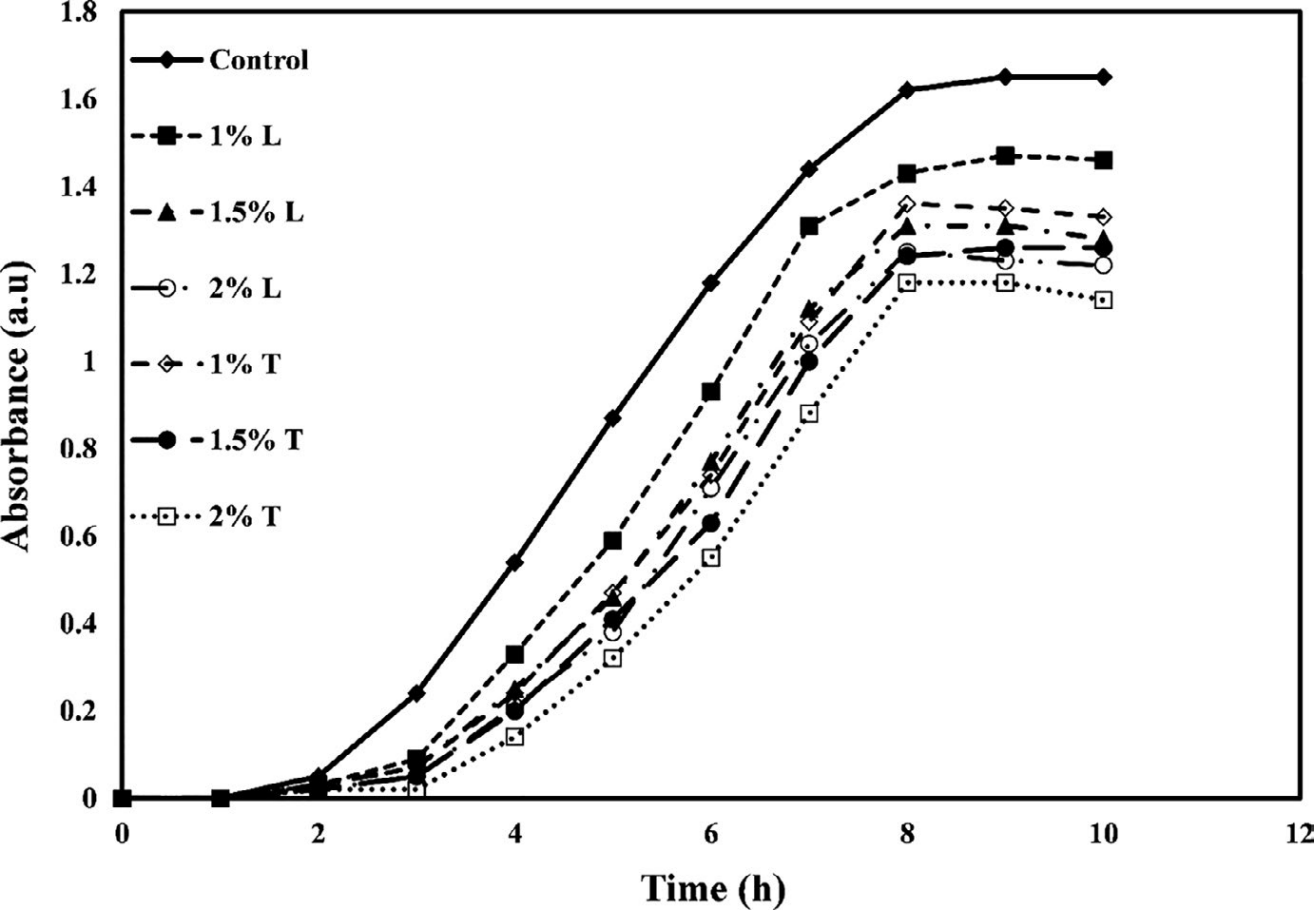
*Saccharomyces cervicea* growth kinetic versus PE films containing different levels of thymol (T) or linalool (L)

The growth curves of *E. coli, Staphylococcus aureus*, *Listeria innocua,* and *Saccharomyces cervicea* are shown in Figures 1, 2, 3 and 4, respectively. As shown in all four figures, the use of different levels of linalool and thymol in PE films increased the lag phase and reduced the proliferation of bacteria and mold in the logarithmic phase and the faster death of these microorganisms. Both active compounds studied in this study showed effective and significant antimicrobial activity, but the activity of thymol was observed higher than linalool.

The study also found that gram‐positive bacteria were more sensitive to antimicrobial compounds (thymol and linalool) than gram‐negative bacteria. Higher antimicrobial activity of most extracts and essential oils against gram‐positive bacteria than gram‐negative bacteria is due to structural differences in the cytoplasmic membrane and cell wall of gram‐positive bacteria than gram‐negative bacteria so that gram‐negative bacteria have a universal cell membrane that, unlike gram‐positive bacteria, prevents the diffusion of hydrophobic compounds into liposaccharide coating (Oraki et al., 2011). Acevedo‐Fani et al. (2015) also found that the gram‐positive bacterium *Bacillus subtilis* was more sensitive to the thymol than the gram‐negative bacterium *E. coli*.

The mechanisms of antimicrobial action of thymol have been found in bacterial membrane destruction, leakage of intracellular compounds, and, finally, bacterial cell death. This active compound can also block the operation of diffusion pumps, prevent cell motility, and prevent the membrane ATPase enzymes (Kachur & Suntres, 2020). Li et al. (2020) reported that gelatin films containing thymol nanoemulsions showed a significant antimicrobial effect against gram‐positive and gram‐negative bacteria and exerted this long‐term antimicrobial activity.

Researchers have shown that linalool can destroy the integrity of cell membranes and increase membrane permeability and control the leakage of cell substances such as nucleic acid. Linalool can also reverse the polarization of cellular membrane and disrupt cellular metabolic activity, thereby causing microbial cell death (Liu et al., 2020). Prakash et al. (2019) also reported that linalool caused the death of *Salmonella thyphimurium* cells by destroying cellular membranes and leaking intracellular substances.

In a similar study, Del Nobile et al. (2008) examined the effect of thymol‐containing zein film on *Bacillus cereus, Streptococcus thermophiles,* and *Pseudomonas* by drawing a growth/survival curve in a liquid medium and showed that antibacterial effects of film containing thymol are a function of time. By increasing time, the antibacterial activities also increase. Other researchers have found that films containing peppermint, thyme, lemon, and cinnamon essential oils, and grape and garlic extracts inhibit the growth of *E. coli* (Emiroğlu et al., 2010; Rojas‐Graü et al., 2007; Sivarooban et al., 2008). Mehdizadeh et al. (2012) studied the antimicrobial activity of starch–chitosan composite films containing Ziziphora essential oil and reported that the antimicrobial effects increased significantly with increasing essential oil concentration.

### 3.2 Effects of PE films containing thymol and linalool on the microbial spoilage of mozzarella cheese

#### 3.2.1 Mold and yeast counts in mozzarella cheese samples

**FIGURE 5:**
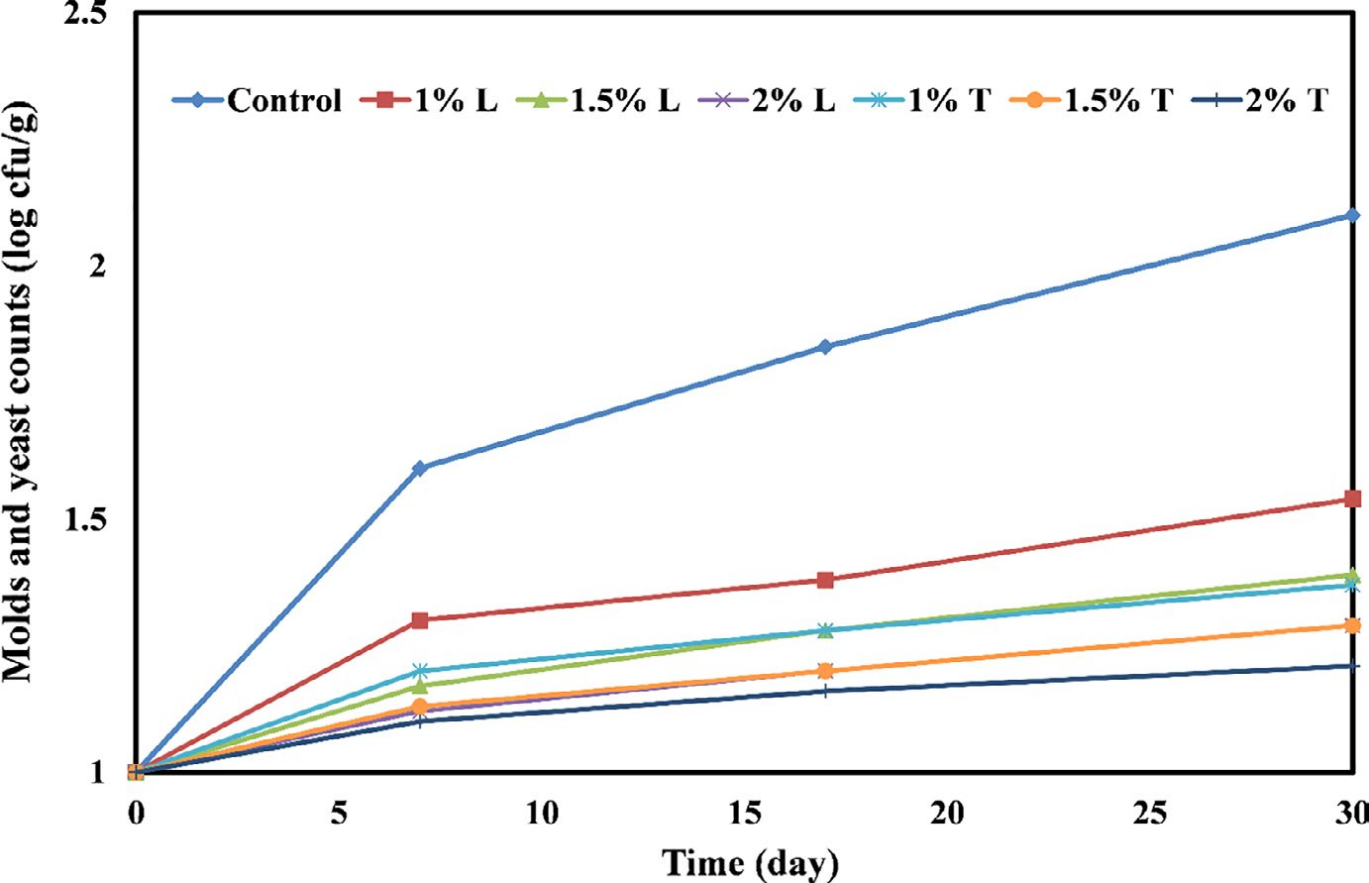
Changes in the number of molds and yeast in mozzarella cheeses packed with PE films containing different levels of thymol (T) or linalool (L)

The changes in the number of molds and yeasts in the control cheese and cheeses packed in PE films containing different levels of thymol and linalool are shown in Figure 5. As the figure shows on the first day, there was not a difference between the number of molds and yeasts in different cheese samples, and the number of these microorganisms in different cheese samples was 1.00 Log CFU/g. Due to the growth and proliferation of microorganisms during the storage period, the number of molds and yeasts in all cheese samples increased significantly (*p* < .05). The highest growth rate of molds and yeasts was observed in the control sample, so that in this sample, from the first day to the 30 days of storage, the number of molds and yeasts increased from 1.00 to 2.10 Log CFU/g. The lowest mold and yeast multiplication rate was observed in cheeses packed in PE film containing 2% thymol, which increased from 1.00 to 1.21 Log CFU/g during the storage period. In general, the number of molds and yeasts decreased significantly with the increasing concentration of linalool and thymol in PE films (*p* < .05).

#### 3.2.2 *E. coli* and *Staphylococcus aureus* count in mozzarella cheese samples

The study results of the presence of gram‐negative *E. coli* and gram‐positive *Staphylococcus aureus* in low‐moisture mozzarella cheese samples are given in Tables 1 and 2, respectively. These results show that *E. coli* and *Staphylococcus aureus* was not found in the control sample until the 7th day, but on the 17th day these bacteria were observed in control (17.00 and 32.05 Log CFU/g, respectively), and their numbers increased significantly until the 30th day of storage (25.10 and 49.23 Log CFU/g, respectively) (*p* < .05). However, until the last day of storage, cheeses packed in PE films containing different levels of thymol and linalool did not have *E. coli* and *Staphylococcus aureus*.

**TABLE 1:** Comparison of the *Escherichia coli* numbers (Log CFU/g) in packaged mozzarella cheese samples in active PE films during storage

Samples	Day 0	Day 7	Day 17	Day 30
Control	ND	ND	17.00 ± 0.03 b	25.10 ± 0.09 a
1% Linalool	ND	ND	ND	ND
1.5% Linalool	ND	ND	ND	ND
2% Linalool	ND	ND	ND	ND
1% Thymol	ND	ND	ND	ND
1.5% Thymol	ND	ND	ND	ND
2% Thymol	ND	ND	ND	ND

Note

Values represent mean (*n* = 3) ± *SD*. Different letters in each column represent significant differences at 5% level of probability among sample cheeses.

**TABLE 2:** Comparison of the *Staphylococcus aureus* numbers in packaged mozzarella cheese samples in active PE films during storage

Samples	Day 0	Day 7	Day 17	Day 30
Control	ND	ND	32.05 ± 0.08 b	49.23 ± 0.13 a
1% Linalool	ND	ND	ND	ND
1.5% Linalool	ND	ND	ND	ND
2% Linalool	ND	ND	ND	ND
1% Thymol	ND	ND	ND	ND
1.5% Thymol	ND	ND	ND	ND
2% Thymol	ND	ND	ND	ND

Note

Values represent mean (*n* = 3) ± *SD*. Different letters in each column represent significant differences at 5% level of probability among sample cheeses.

In active packaging systems, the antimicrobial agent is gradually transferred from the coating layer to the food during storage. Therefore, by increasing the concentration of the antimicrobial agent in the active packaging film, the food product becomes safer against microorganisms (Cagri et al., 2002). In general, due to the hydrophobic nature of the active chemical compounds of plant essential oils, these compounds change the membrane of bacteria and mitochondrial cells and make their structure permeable (Gyawali & Ibrahim, 2014). The chemical compounds in the essential oils can also hydrolyze bacteria enzymes and lead to the loss of protein stimulus, electron flow, active displacement, and coagulation of cell substances (Shahbazi et al., 2016). Although a certain amount of leakage of intracellular compounds (as a result of membrane damage) from bacteria cells can be tolerated without losing the possibility of life, continued leakage of cellular substances or release of vital molecules and ions will lead to bacteria death (Jouki et al., 2014).

In a study of the effect of chitosan–starch film on the shelf life of Mongolian cheese, Mei et al. (2013) reported that the use of this coating was effective in controlling the microbial load of cheese. These researchers showed that this film on cheese samples delayed the growth of mold in packaged cheeses. Kavas and Kavas (2014) observed that the number of *E. coli*, molds, and yeasts in cheese samples decreased significantly with increasing levels of peppermint essential oil in whey protein isolate coating, so that, during the storage period in the control cheese, the number of molds and yeasts increased, but in cheeses coated with a solution containing peppermint essential oil, the colony number gradually decreased, which was due to the antimicrobial activity of this essential oil. In another study, it was shown that with increasing the concentration of *Mentha Piperita* L. in the Bojnourd local cheese, the number of molds, yeasts, and coliforms in cheese samples decreased significantly (Noghani & Sameti, 2015).

## 4 CONCLUSION

In this study, the antimicrobial activity of thymol or linalool in PE films was investigated, and then production films were used for packaging mozzarella cheese. Based on the results, both thymol and linalool showed good antimicrobial activity in PE films and significantly reduced the growth and proliferation rate of the microorganisms studied in this research. The use of PE films containing different concentrations of thymol or linalool also prevented the growth of *E. coli* and *S. aureus* in mozzarella cheese during refrigeration. Furthermore, they controlled the growth of molds and yeast in cheese samples. Therefore, the PE films containing at least 2% thymol or linalool can be used as desirable antimicrobial packaging in the dairy industry. Further studies are recommended to investigate the release kinetics of linalool and thymol from different PE films.

## CONFLICT OF INTEREST

The authors declare no conflict of interest.

## AUTHOR CONTRIBUTION

**Shadi Chang:** Data curation (equal); Formal analysis (equal); Resources (equal); Validation (equal); Visualization (equal); Writing‐original draft (equal). **Abdorreza Mohammadi Nafchi:** Conceptualization (equal); Resources (equal); Supervision (equal); Validation (equal); Writing‐review & editing (equal). **Homa Baghaie:** Data curation (equal); Formal analysis (equal); Methodology (equal); Supervision (equal).

## ETHICAL APPROVAL

This study does not involve any human or animal testing.

## Data Availability

The data that support the findings of this study are available from the corresponding author, upon reasonable request.
